# Characterization of Chicken α_2A_-Adrenoceptor: Molecular Cloning, Functional Analysis, and Its Involvement in Ovarian Follicular Development

**DOI:** 10.3390/genes13071113

**Published:** 2022-06-21

**Authors:** Biying Jiang, Baolong Cao, Zhichun Zhou, Zejiao Li, Can Lv, Jiannan Zhang, Heyuan Zhang, Yajun Wang, Juan Li

**Affiliations:** Key Laboratory of Bio-Resources and Eco-Environment of Ministry of Education, College of Life Sciences, Sichuan University, Chengdu 610065, China; v_jby86@163.com (B.J.); cbl-scu@outlook.com (B.C.); 15111057261@163.com (Z.Z.); lzejiao7077@163.com (Z.L.); 18202860297@163.com (C.L.); biozhangjn@scu.edu.cn (J.Z.); zhy_tardigrata@163.com (H.Z.); cdwyjhk@163.com (Y.W.)

**Keywords:** chickens, α_2_-adrenoceptor, *ADRA2A*

## Abstract

Adrenoceptors are suggested to mediate the functions of norepinephrine (NE) and epinephrine (EPI) in the central nervous system (CNS) and peripheral tissues in vertebrates. Compared to mammals, the functionality and expression of adrenoceptors have not been well characterized in birds. Here, we reported the structure, expression, and functionality of chicken functional α_2A_-adrenoceptor, named *ADRA2A*. The cloned chicken *ADRA2A* cDNA is 1335 bp in length, encoding the receptor with 444 amino acids (a.a.), which shows high amino acid sequence identity (63.4%) with its corresponding ortholog in humans. Using cell-based luciferase reporter assays and Western blot, we demonstrated that the ADRA2A could be activated by both NE and EPI through multiple signaling pathways, including MAPK/ERK signaling cascade. In addition, the mRNA expression of *ADRA2A* is found to be expressed abundantly in adult chicken tissues including thyroid, lung, ovary and adipose from the reported RNA-Seq data sets. Moreover, the mRNA expression of *ADRA2A* is also found to be highly expressed in the granulosa cells of 6–8 mm and F5 chicken ovarian follicles, which thus supports that ADRA2A signaling may play a role in ovarian follicular growth and differentiation. Taken together, our data provide the first proof that the α_2A_-adrenoceptor is functional in birds involving avian ovarian follicular development.

## 1. Introduction

Epinephrine (EPI) and norepinephrine (NE), known as catecholamines, play an important role in the central nervous system and peripheral tissues. Circulating EPI is derived from chromaffin cells of the adrenal medulla, which acts on other cells through an endocrine route. Most NE is released from sympathetic nerves that act directly through synapses as a neurotransmitter, while a few NE originate from the adrenal medulla functioning as a circulating hormone [1]. As stress hormones, both NE and EPI are involved in many physiological processes, including increased heart rate and blood pressure, the contraction of skeletal muscle, glycogenolysis and lipolysis, etc. [2,3,4,5,6]. Furthermore, they participate in arousal, emotion, memory, circadian rhythm, regulation of hormone release and ovarian function [7,8,9,10]. Due to their significant roles in physiologic processes, the dysfunction of NE and EPI systems may cause some disorders, such as pheochromocytoma, attention deficit hyperactivity disorder (ADHD), sympathetic hyperactivation, and autonomic failure [11,12,13,14].

The functions of NE and EPI are mediated by adrenoceptors (or adrenergic receptors), which belong to the class A G-protein-coupled receptors (GPCRs) [15]. According to their differences in pharmacological profiles and functions, the adrenoceptors have been classified into three groups in mammals, with a total of nine subtypes, including: α_1_-adrenoceptors (α_1A_, α_1B_ and α_1D_-adrenoceptors), α_2_-adrenoceptors (α_2A_, α_2B_ and α_2C_-adrenoceptors) and β-adrenoceptors (β_1_, β_2_ and β_3_-adrenoceptors) [16,17]. The α_2_-adrenoceptors are characteristic for the activation of the compounds B-HT920, B-HT933 and UK-14304, or of the compound α-methylNA, which are competitively inhibited by low concentrations of yohimbine, rauwolscine or idazoxan [17]. In line with the typical signaling pathway of GPCR, the α_2-_adrenoceptors are reported to bind to G_i/o_ protein, thereby inhibiting adenylyl cyclase (AC) and decreasing cAMP accumulation in mammals [18,19]. In addition to the coupling to G_i_ protein, the α_2_-adrenoceptors signaling pathway also includes the subsequent involvement of βγ subunits in PLC activation, thus activating MAPK/ERK signaling pathways and Ca^2+^ mobilization [20,21,22].

As in mammals, NE and EPI bind their adrenoceptors, also playing a vital role in non-mammalian vertebrates. In birds, NE and EPI have been reported to be associated with aggression, stress response, lipid metabolism, glycogenolysis, circadian rhythm, food intake, pecking behavior and production potential [23,24,25,26]. Similar to the detection in mammals, innervation has also been detected in avian ovaries [9]. In chickens, high-performance liquid chromatography and electrochemical detection revealed the high concentration of NE in the cranial part of the chicken ovary [27]. NE or EP significantly increase the ovulation rate and the plasma concentration of estradiol-17β, progesterone, etc. [28,29,30]. In contrast with the clear α-adrenoceptor subtype to be identified in mammalian species mediating function [31,32,33,34,35], the potential α-adrenoceptor subtype involving chicken ovarian function remains to be identified.

In the present study, we reported the complete cDNA sequence of the chicken α_2A_-adrenoceptor and identified its signaling properties and distribution among chicken tissues. In combination with the findings of the high abundance of *ADAR2A* mRNA in chicken ovary, the relative abundance of *ADAR2A* mRNA in growing follicles was also investigated. Our study supports the hypothesis that NE could induce the proliferation and differentiation of granulosa cells from chicken prehierarchical follicles, thus establishing the basis to explore the role of NE, EPI and their receptors in the ovary of birds and shedding more light on their physiological functions among vertebrates.

## 2. Materials and Methods

### 2.1. Ethics Statement

All the animal experiments were conducted in accordance with the Guidelines for Experimental Animals issued by the Ministry of Science and Technology of the People’ s Republic of China. The experimental protocol was approved by the Animal Ethics Committee of College of Life Sciences, Sichuan University, China, and the assurance number is 20210308008 (8 March 2021).

### 2.2. Chemicals, Antibodies and Primers

All chemicals were obtained from Sigma-Aldrich (St. Louis, MO, USA), and restriction enzymes were obtained from Takara Biotechnology Co., Ltd. (Dalian, China). All primers used in this study were synthesized by Beijing Genome Institute (BGI) and are listed in Table 1. Norepinephrine and epinephrine were purchased from Aladdin Bio-Chem Technology Co., Ltd. (Shanghai, China) and Aike Reagent Co., Ltd. (Chengdu, China), respectively. Antibodies for β-actin (#4970), phosphorylated CREB (#9198) and phosphorylated ERK1/2 (#9101) were purchased from Cell Signaling Technology (CST, Bevely, MA, USA). The pharmacological agents including H89, U73122, PD98059 and 2-aminoethoxydiphenyl borate (2-APB) were purchased from Calbiochem (Merck KGaA, Darmstadt, Germany). The EdU Cell Proliferation Kit was purchased from RiboBio Co., Ltd. (Guangzhou, China).

### 2.3. Total RNA Extraction

For chicken tissue RNA extraction, adult chickens (1-year-old) of Lohmann layer strain were purchased from MUXING company in Sichuan, China. Eight adult chickens (4 males and 4 females) were sacrificed, and different tissues including the hypothalamus were collected for RNA extraction. All samples were stored at −80 °C before use. 

For chicken granulosa cell RNA extraction, the laying hens with normal follicle hierarchies were purchased from the local company as described above. In total, four hens (*n* = 4) were subjected to follicle collection. Following the method described by Zhu et al. [36], the white follicles with 1–3, 3–5, and 6–8 mm in diameter, which belong to the follicles not being selected, were collected. The F5 follicles, which belong to the small yellow follicles after selection with the diameter ranging from 11–12 mm, were also collected. The large preovulatory follicles (F3, F1 follicle) directly before ovulation with diameters ranging from 34–42 mm were also collected in the present study. Each follicle was employed for RNA extraction independently. Briefly, the follicle was soaked in 1xPBS solution buffer at 4 °C. Based on the method described [37], as completely as possible, the granulosa cells and the theca cells were isolated and dispersed in RNAzol reagent (Molecular Research Center, Cincinnati, OH, USA). For RNA extraction, total RNA from the tissues and the ovarian follicles was extracted with RNAzol reagent (Molecular Research Center, Cincinnati, OH, USA) according to the manufacturer’ s instructions, which was then diluted in H_2_O treated with diethylpyrocarbonate (DEPC). Briefly, after precipitating with DEPC-treated ultra-pure water, the RNAzol lysed tissues (or follicle samples) were centrifuged (12,000 rpm, 10 min). BAN solution (4-bromoanisole) was added to purify the RNA and to eliminate genomic DNA. Then, an equal volume of cold 100% isopropanol was added. The precipitated pellet was washed three times with 600 μL 75% ethanol and dissolved in 30 μL RNase-free water. The approximate concentration and purity of samples were determined using a Onedrop1000 Spectrophotometer. 

### 2.4. Expression Analyses of ADRA2A in Chicken Tissues and Developmental Follicles

High-throughput transcriptome analysis now serves as a powerful technique to reveal differential gene expression. In the present study, the three RNA-Seq data sets deposited in GenBank were employed to reveal the expression abundance of *ADRA2A* among chicken tissues and the developmental follicles. One RNA-Seq data set from red jungle fowl (SRP016501) was established by Burge et al. in an effort to reveal the evolutionary dynamics of gene regulation across vertebrates [38]. Another RNA-Seq data set (ERP014416) was deposited by the Roslin Institute on 21 tissues collected from 9 female J-line chickens (16–17 weeks). The third RNA-seq data set is from our reported RNA-seq data (GSE112470), which was collected from adult chicken ovarian follicles (6–8 mm, F5 and F1 follicles) [36]. In the present study, based on the three RNA-Seq data sets, we quantified the gene expression level using the transcript quantitative analysis tool Salmon v0.10.2 and using default parameters. The relative abundance of chicken *ADRA2A* transcripts was expressed as transcripts per million (TPM).

### 2.5. Reverse Transcription and Quantitative Real-Time PCR

For reverse transcription, total RNA (2 μg) and 0.5 μg of oligo-deoxythymidine were mixed in a volume of 5 μL, incubated at 70 °C for 10 min, and cooled at 4 °C for 2 min. Then, the first strand buffer, 0.5 mM each deoxynucleotide triphosphate (dNTP) and 100 U moloney murine leukemia virus (MMLV) reverse transcriptase (Takara) were added into the reaction mix in a total volume of 10 μL. Reverse transcription (RT) was performed at 42 °C for 90 min. After the reaction, the cDNA templates were diluted with MilliQ-H_2_O and stored at −20 °C.

According to our previously established method [39], quantitative real-time PCR was performed to examine the expression of *ADRA2A* mRNA among developmental ovarian follicles. In addition, the expression of the gene encoding steroidogenic acute regulatory protein (*STAR*), which mediates the rate-limit step in steroid hormone synthesis [40], and the gene encoding the inhibitor of differentiation/DNA binding family member 3 (*ID3*), which plays a key role in follicle selection [41,42] were also examined by quantitative real-time PCR in the present study. 

The qPCR primers of chicken *ADRA2A* (XM_004942276.2), *STAR* (NM_204686.2) and *ID3* (NM_204589.1) were designed based on sequences in GenBank and are listed in Table 1. The primers (10 μM), dNTP (10 mM), Easy Taq Buffer, Easy Taq DNA polymerase (TransGen Biotech), Eva Green (Biotium), MilliQ-H_2_O and templates were mixed in a total volume of 20 μL. Then, the reaction mix was conducted on the CFX96 Real-time PCR Detection System (Bio-Rad). The amplification conditions included an initial denaturation for 10 min at 94 °C followed by 20 s denaturation at 94 °C, 15 s annealing at 57 °C (*STAR*)/60 °C (*ID3*), and 30 s extension at 72 °C for 40 cycles.

### 2.6. Cloning the cDNA of Chicken ADRA2A

Based on the predicted cDNA sequences of chicken *ADRA2A* (XM_004942276.2) deposited in GenBank, gene-specific primers were designed to amplify the complete ORF of *cADRA2A* from adult chicken hypothalamus tissue. The amplified PCR products were cloned into the pcDNA3.1(+) vector (Invitrogen) and subjected for sequence (BGI).

### 2.7. Sequence Alignment and Synteny Analysis

The protein sequences of the *ADRA2A* gene in several vertebrates based on the evolutionary background, including *Gallus* (chicken, NP_997520), *Homo sapiens* (human, NP_000672), *Mus musculus* (mouse, NP_031443), *Pelodiscus sinensis* (Chinese softshell turtle, XP_006135062), *Xenopus tropicalis* (Western clawed frog, NP_001072843) and *Danio rerio* (zebrafish, NP_997520) were accessed from the NCBI public database. The deduced amino acid sequences were aligned using the ClustalW program (BioEdit, Carlsbad, CA, USA) [43]. The putative transmembrane (TM) domains were predicted by using an online protein topology prediction tool DeepTMHMM, which is a protein structure prediction tool that uses deep learning algorithms to predict transmembrane structural domains [44]. To determine whether the cloned chicken *ADRA2A* is orthologous to *ADRA2A* identified in other vertebrate species, the neighboring genes of *ADRA2A* in genomic regions of chicken and other vertebrate species were examined, which was formatted using a genome browser Genomicus available online at https://www.genomicus.bio.ens.psl.eu/genomicus-93.01/cgi-bin/search.pl (accessed on 13 October 2021) [45].

### 2.8. Functional Characterization of Chicken ADRA2A

According to our previously established methods [46,47,48], cADRA2A was transiently expressed in CHO cells and treated by NE (10^−10^ to 10^−4^ M) or EPI (10^−10^ to 10^−4^ M). The receptor-activated signaling pathways were then examined by pGL3-CRE–luciferase, pGL4-SRE–luciferase and pGL3-NFAT–luciferase reporter systems, which were capable of monitoring cAMP/PKA, MAPK/ERK signaling pathways and intracellular calcium, respectively. In brief, CHO cells were cultured on a six-well plate (Nunc, Roskilde, Denmark) and grown for 24 h before transfection. The cells were then transfected with a mixture containing 700 ng pGL3-CRE–luciferase reporter construct (or pGL4-SRE–luciferase or pGL3-NFAT-RE–luciferase), 200 ng of receptor expression plasmid (or empty pcDNA3.1 vector as a negative control), 20 ng of pRL-TK construct (containing a Renilla luciferase gene, used as an internal control), and 2 μL jetPRIME transfection reagent (Polyplus Transfection, Illkirch, France) in 200 μL buffer. Twenty-four hours later, CHO cells were sub-cultured into a 96-well plate at 37 °C for an additional 24 h before treatment. After removal of medium from the 96-well plate, the cells were treated with 100 μL NE/EPI-containing medium (or NE/EPI-free medium) for 6 h. Finally, CHO cells were lysed with 1× passive lysis buffer for luciferase assay (Promega), and the luciferase activity of the cell lysate was measured by a Multimode microplate Reader (TriStar LB941, EG&G Berthold, Germany) according to the manufacturer’s instruction.

### 2.9. Cell Proliferation Assay 

Chicken granulosa cells isolated from 6–8 mm follicles were cultured in 96-well cell plates for 4–6 h, as previously reported [49]. Then, the granulosa cells were treated by NE (10 μM) and EdU (20 μM) for 16 h. EdU is a kind of thymidine analogue, which can replace thymine in the cell proliferation period and infiltrate into the replicating DNA molecule. After 16 h treatment, cells were fixed in 4% paraformaldehyde and washed with PBS. Then, the cells were treated by 2 mg/mL glycine and washed with PBS. Cells were treated by 0.1% Triton-X-100 for 10 min for permeabilization. After fixation and permeabilization, cells were incubated with Apollo^®^ stain reaction solution for 30 min to react with EdU specifically to detect DNA replication activity. Cells were then incubated with Hoechst33342 for 30 min for nuclear staining. EdU-positive signal is the red fluorescence that can completely overlap with the blue fluorescence of Hoechst33342 staining signal, suggesting the proliferation ability of these granulosa cells.

### 2.10. Western Blot

As described in our previous studies [48,50], CHO cells transfected with cADRA2A expression plasmid were cultured on a 24-well plate at 37 °C and then treated by NE (10 μM) for 10 min. Then, the cells were lysed and used for Western blot detection of β-actin, phosphorylated CREB (pCREB) (43 kDa) and (or) ERK1/2 (pERK1/2) (44/42 kDa). 

To examine whether cADRA2A could be induced by NE in cultured granulosa cells, the chicken granulosa cells isolated from 6–8 mm follicles were cultured in 48-well cell plates for 6 h and treated by NE (10 μM) for 10 min. Then, cells were lysed and used for Western blot detection of phosphorylated ERK2 as mentioned above.

### 2.11. Data Analysis

The relative mRNA levels of *ADRA2A* were first calculated as the ratios to that of β-actin and then expressed as the percentage (or fold change) compared to their respective controls (chosen tissues). Semi-quantitative analysis of band intensity from Western blot was performed using the Image J program (Image J software; National Institutes of Health), and the relative protein levels normalized by that of intracellular β-actin were then expressed as the percentage compared with respective controls (without treatment). The data were analyzed by Student’s *t* test (between two groups) in GraphPad Prism 7 (GraphPad Software, San Diego, CA, USA). To validate our results, all experiments were repeated at least twice.

## 3. Results

### 3.1. Cloning of Chicken ADRA2A

**Figure 1:**
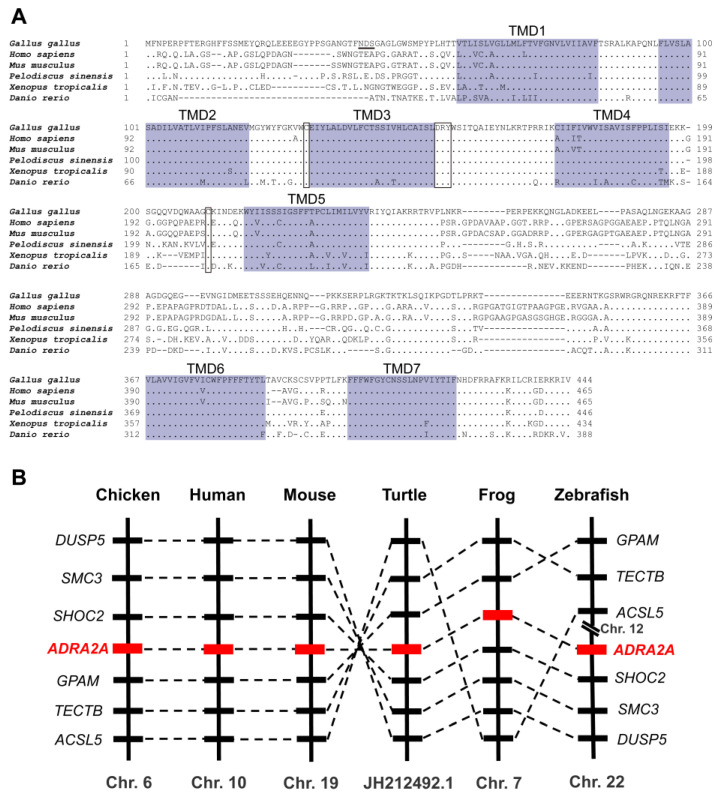
(**A**) Amino acid alignment of chicken (*Gallus gallus*) ADRA2A with that of human (*Homo sapiens*), mouse (*Mus musculus*), turtle (*Pelodiscus sinensis*), frog (*Xenopus tropicalis*), and zebrafish (*Danio rerio*). The seven transmembrane domains (TMD1–7) are shaded; the conserved ‘DRY’ motif and cysteine (C) residues for disulfide bond formation are boxed; the potential N-glycosylation sites (NXS, X represents any amino acid residue except proline) are underlined. (**B**) Synteny analysis showing that *ADRA2A* was located in distinct syntenic regions conserved in chicken (*Gallus gallus*), human (*Homo sapiens*), mouse (*Mus musculus*), turtle (*Pelodiscus sinensis*), frog (*Xenopus tropicalis*), and zebrafish (*Danio rerio*).

Based on the predicted cDNA sequences of *cADRA2A* (XM_004942276.2), we cloned the full-length cDNA of the *cADRA2A* gene from the adult chicken hypothalamus. The cloned c*ADRA2A* cDNA is 1335 bp in length and encodes a receptor of 444 amino acid. Sequence analysis revealed that it showed high amino acids sequence identity with ADRA2A of *Homo sapiens* (63.4%), *Mus musculus* (63.1%), *Pelodiscus sinensis* (85.1%), *Xenopus tropicalis* (72.4%), and *Danio rerio* (61.2%). As in mammals, chicken ADRA2A showed the typical seven transmembrane domains as G-protein coupled receptors, a DRY motif that functions for G-protein coupling [51]. As shown in Figure 1A, the two cysteines for disulfide bond formation could be detected. In addition, the potential N-glycosylation sites (NDS) were underlined. Using synteny analysis, we found that *ADRA2A* exists in chickens and other examined species including human (*Homo sapiens*), mouse (*Mus musculus*), turtle (*Pelodiscus sinensis*), frog (*Xenopus tropicalis*), and zebrafish (*Danio rerio*) (Figure 1B). Moreover, some genes adjacent to *ADRA2A* were consistent with these species examined, indicating that chicken *ADRA2A* was orthologous to the *ADRA2A* genes of humans and other species.

### 3.2. Functional Analyses of Chicken ADRA2A

To investigate the functionality of the chicken ADRA2A receptor, the ADRA2A receptor was transiently expressed in CHO cells and treated with NE and EPI. Receptor activation was examined by pGL3-CRE–luciferase reporter, pGL4-SRE–luciferase reporter, and pGL3-NFAT–luciferase reporter systems. 

**Figure 2:**
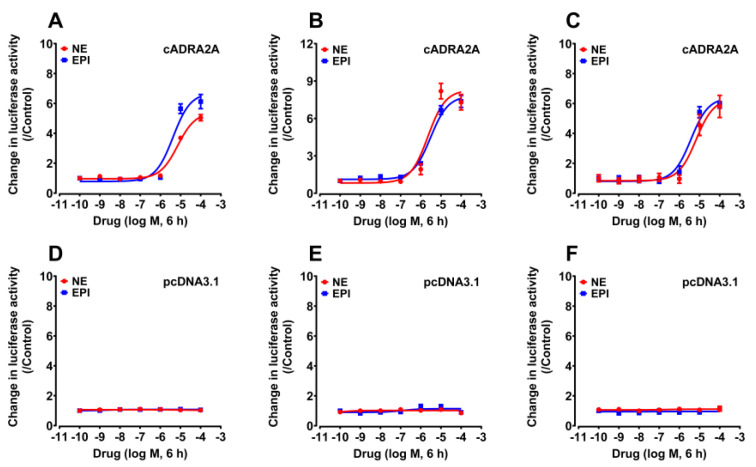
Effects of NE and EPI on activating chicken ADRA2A expressed in CHO cells. (**A**–**C**) Activation of cADRA2A expressed in CHO cells upon NE (or EPI) treatment, monitored by pGL3-CRE–luciferase (**A**), pGL4-SRE–luciferase (**B**), and pGL3-NFAT–luciferase (**C**) reporter systems. (**D**,**E**) Effects of NE and EPI on activating luciferase activity of CHO cells transfected with empty pcDNA3.1 vector, monitored by pGL3-CRE–luciferase (**D**), pGL4-SRE–luciferase (**E**), and pGL3-NFAT–luciferase (**F**) reporter systems. Each data point represents the mean ± SEM of three replicates (*n* = 3). Each dose–responsive curve shows one representative experiment (repeated three times).

As shown in Figure 2A, NE and EPI could dose-dependently stimulate luciferase activity of CHO cells via activation of cADRA2A with similar EC_50_ values of 7.21 ± 1.15 and 4.23 ± 1.36 μM, using the pGL3-CRE–luciferase reporter system. The activation of cADRA2A indicated that it is a functional receptor for NE and EPI, and its activation could stimulate the cAMP/PKA signaling pathway. Using the pGL4-SRE–luciferase reporter system, we demonstrated that cADRA2A could potently be activated by NE (2.26 ± 0.97 μM) and EPI (3.04 ± 0.64 μM). The findings suggested that the cADRA2A is functional and can be coupled to the MAPK/ERK signaling cascade (Figure 2B). As monitored by the pGL3-NFAT–luciferase reporter system, we found that cADRA2A could be potently activated by NE and EPI with similar potencies (6.74 ± 2.64 and 3.73 ± 1.18 μM) (Figure 2C, Table 2). The findings suggest that activation of cADRA2A could also trigger calcium mobilization.

In parallel to the above experiments, peptide treatment (10^−10^ to 10^−4^ M) did not alter luciferase activity of CHO cells transfected with the empty pcDNA3.1 vector, confirming the specific action of the peptide on receptor activation (Figure 2D–F).

**Figure 3:**
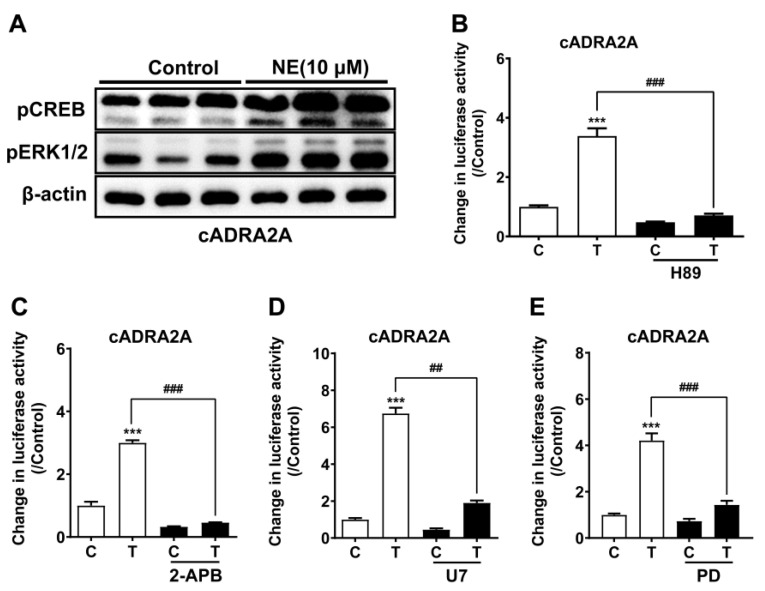
(**A**) Western blot showing that NE treatment (10 μM, 10 min) could enhance the phosphorylation levels of ERK1/2 (pERK1/2) in CHO cells expressing chicken ADRA2A. In parallel, NE treatment could also enhance the phosphorylation levels of CREB (pCREB) in CHO cells expressing chicken ADRA2A. Each experiment was repeated three times (*n* = 3). Each figure shows one representative experiment. (**B**) Effects of H89 (10 μM) on NE (10 μM, 6 h)-induced luciferase activity of CHO cells expressing cADRA2A, monitored by pGL3-CRE–luciferase reporter system. (**C**) Effects of 2-APB (100 μM) on NE (10 μM, 6 h)-induced luciferase activity of CHO cells expressing cADRA2A, monitored by pGL3-NFAT–luciferase reporter system. (**D**) Effects of U73122 (U7, 20 μM) on NE (10 μM, 6 h)-induced luciferase activity of CHO cells expressing cADRA2A, monitored by pGL4-SRE–luciferase reporter system. (**E**) Effects of PD98059 (PD, 100 μM) on NE (10 μM, 6 h)-induced luciferase activity of CHO cells expressing cADRA2A, monitored by pGL4-SRE–luciferase reporter system. Each drug was added 0.5 h before NE treatment. In each graph, ‘T’ represents peptide treatment and ‘C’ represents control without peptide treatment. Each data point represents the mean ± SEM of four replicates (*n* = 4). Each figure shows one representative experiment (repeated three times). The data were analyzed by Student’s *t* test. ***, *p* < 0.001 vs. control (in the absence of drugs); ^###,^ *p* < 0.001 vs. peptide treatment (in the absence of drug), ^##,^ *p* < 0.01 vs. peptide treatment (in the absence of drugs).

Using Western blot, we demonstrated that NE (10 μM) treatment could enhance CREB (43 kDa) phosphorylation and ERK1/2 (44/42 kDa) phosphorylation in CHO cells expressing cADRA2A, which further supported the functional coupling of cADRA2A with cAMP/PKA and MAPK/ERK signaling cascades (Figure 3A).

To verify the functional coupling of cADRA2A to cAMP/PKA, MAPK/ERK and intracellular calcium mobilization, pharmacological drugs targeting the three signaling pathways were used. As shown in Figure 3B–E, H89 (a PKA inhibitor,10 μM), 2-APB (an inhibitor of IP_3_ receptor, which blocks IP_3_-induced calcium mobilization, 100 μM), U73122 (a phospholipase C inhibitor, 20 μM), and PD98059 (an inhibitor of MEK/ERK signaling cascade, 100 μM) could significantly inhibit NE (10 μM, 6 h)-induced luciferase activities of CHO cells expressing cADRA2A. The findings further support the coupling of cADRA2A to the cAMP/PKA/CREB signaling pathway, the MAPK/ERK signaling cascade and calcium mobilization.

### 3.3. Expression of ADRA2A in Chicken Tissues 

**Figure 4:**
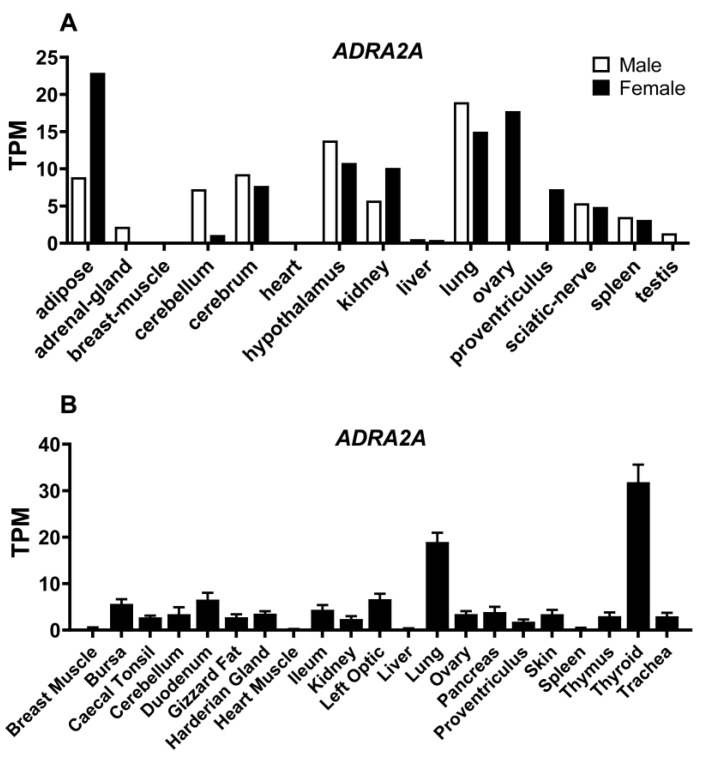
RNA-Seq data showing the expression of *ADRA2A* in red jungle fowl and chicken J-line. (**A**) Expression of *ADRA2A* mRNA from red jungle fowl (SRP016501). (**B**) Expression of *ADRA2A* mRNA from chicken J-line (ERP014416).

In the present study, the two RNA-Seq data sets deposited into the public database were employed to reveal the expression levels of *ADRA2A* among chicken tissues. As shown in Figure 4A, the expression of *ADRA2A* mRNA showed a high abundance in the tissues, including ovary, lung and adipose in red jungle fowl (SRP016501). In the central nervous system (CNS), including the hypothalamus, cerebellum and cerebrum, the expression of *ADRA2A* mRNA showed a moderate abundance. In another RNA-Seq data set sampled from chicken J-line (ERP014416), the expression of *ADRA2A* mRNA showed an abundance in lung and thyroid tissues (Figure 4B).

### 3.4. Expression of ADRA2A in Chicken Ovarian Follicles

**Figure 5:**
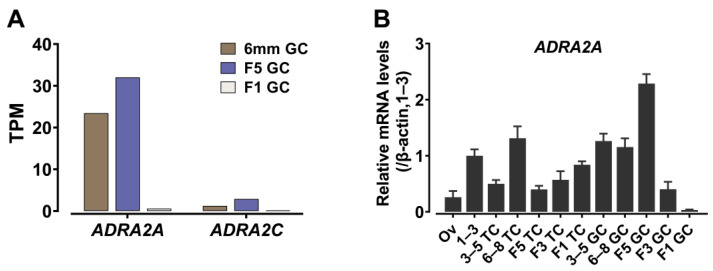
Expression of *ADRA2A* among chicken follicles. (**A**) RNA-Seq data analysis showing the expression of *cADRA2A* among the granulosa cells from chicken follicles (GSE112470). (**B**) qPCR detection of chicken *ADRA2A* mRNA levels in adult chicken ovaries, including the ovarian connective tissue (Ov), 1–3 mm follicle (1–3), theca cells of the follicles with diameter 3–5 mm (3–5 TC), 6–8 mm (6–8 TC), F5 (F5 TC), F3 (F3 TC), F1 (F1 TC), granulosa cells of the follicles with diameter 3–5 mm (3–5 GC), 6–8 mm (6–8 GC), F5 (F5 GC), F3 (F3 GC), F1 (F1 GC). The mRNA levels of each gene were normalized to that of *β-actin* and expressed as the fold difference compared with that of 1–3 mm follicle (1–3). Each data point represents the mean ± SEM of four replicates (*n* = 4). The figure shows one representative experiment (repeated three times).

The high abundance of *ADRA2A* mRNA in the ovary of red jungle fowl was confirmed in this study, in combination with the observance that the ovary is organized as a complex structure with multiple ovarian follicles of varied diameter in hens. In the present study, the expression of *ADRA2A* mRNA was further detected in chicken ovarian follicles. As shown in Figure 5A, based on the RNA-Seq data reported in our lab [36], we investigated the mRNA expression of the *ADRA2A* gene in the granulosa cells of chicken white follicles (6–8 mm), F5 follicles (11–12 mm) and F1 follicles (38–42 mm). We found that the mRNA of *ADRA2A* shows a high expression level in granulosa cells, while the mRNA expression of *ADRA2B*, *ADRA2C* and *ADRA2D* is low or undetectable. 

Since every ovarian follicle is organized as the oocyte with the encircling granulosa cells and theca cells, using the quantitative real-time PCR assay (qPCR), we further examined the mRNA expression of *ADRA2A* in the theca cells and granulosa cells from the follicles: 1–3 mm follicle (1–3), theca cells of the follicles with diameter 3–5 mm (3–5 TC), 6–8 mm (6–8 TC), F5 (F5 TC),F3 (F3 TC) and F1 (F1 TC) follicles, and granulosa cells of the follicles with diameter 3–5 mm (3–5 GC), 6–8 mm (6–8 GC), F5 (F5 GC), F3 (F3 GC), and F1 (F1 GC) follicles. As shown in Figure 5B, there was a high level of *ADRA2A* mRNA expression in 6–8 mm theca cells, with expression decreasing with follicle growth and the selection into the preovulatory stage. In the granulosa cells, *cADRA2A* is highly expressed in the 3–5 mm and 6–8 mm follicles, reaches its peak in the F5 follicles, and then gradually diminishes in the preovulatory follicles.

### 3.5. The Effects of NE on Chicken Ovarian Granulosa Cells

**Figure 6:**
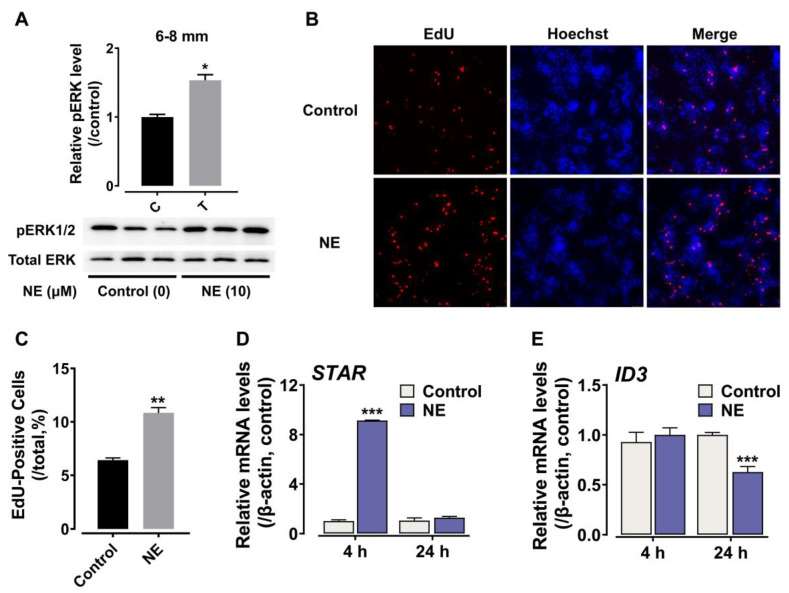
Effect of NE on cultured chicken granulosa cells. (**A**) Western blot detection of NE (10 μM, 10 min) induced phosphorylation levels of ERK2 in cultured 6–8 mm granulosa cells. The data were analyzed by Student’s *t* test. *, *p* < 0.05 vs. control. (**B**) The effects of NE on cell proliferation in cultured granulosa cells from prehierarchical follicles (6–8 mm) of chicken, monitored by EdU incorporation assay. (**C**) Graph showing the relative proportion (%) of EdU-positive cells. Each data point represents the mean ± SEM of three replicates (*n* = 3). The figure shows one representative experiment (repeated two times). The data were analyzed by Student’s *t* test. **, *p* < 0.01 vs. control. (**D**) The effects of NE on the expression of *STAR* in 6–8 mm granulosa cells of chicken. qPCR detection showed that the treatment of NE (10 μM, 4 h) could significantly induce *STAR* expression in cultured 6–8 mm granulosa cells. (**E**) The effects of NE on the expression of *ID3* in 6–8 mm granulosa cells of chicken. qPCR detection showed that the treatment of NE (10 μM, 4 h) could significantly attenuate the expression of *ID3* in cultured 6–8 mm granulosa cells. Each data point represents the mean ± SEM of three replicates (*n* = 3). Each figure shows one representative experiment (repeated three times). The data were analyzed by Student’s *t* test. ***, *p* < 0.001 vs. control.

The high expression of *ADRA2A* in 6–8 mm granulosa cells, together with the previous studies showing that follicle selection is closely related to molecular events in 6–8 mm GC cells, led us to further explore the role of cADRA2A signaling in 6–8 mm granulosa cells. As shown in Figure 6A, NE treatment (10 μM, 10 min) could enhance ERK2 phosphorylation in cultured chicken prehierarchical (6–8 mm) granulosa cells. Accordingly, NE (10 μM, 16 h) could significantly increase the number of EdU-positive cells (Figure 6B,C), indicating that ADRA2A signaling could promote proliferation of granulosa cells in chicken prehierarchical follicles. In addition, as shown in Figure 6D, we found that NE (10 μM, 4 h) could significantly induce *STAR* expression. In the cultured granulosa cells, NE (10 μM, 24 h) also could attenuate the expression of *ID3* (Figure 6E), which encodes the protein as an inhibitor of *FSHR* mRNA transcription, indicating that NE may be involved in the regulation of *FSHR* mRNA transcription prior to follicle selection.

## 4. Discussion

In this study, the cDNA of *ADRA2A* is cloned from chickens. Cell-based luciferase reporter assays and Western blot reveal that the ADRA2A is a functional receptor for NE and EPI. The expression of *ADRA2A* is found to be highly expressed in chicken tissues, including thyroid, ovary, lung and adipose tissues. In addition, in the granulosa cells from chicken developmental follicles including 6–8 mm follicles and F5 follicles, the expression of *ADRA2A* mRNA is also found to be highly expressed. Together with the observance that NE can induce the proliferation of the granulosa cells, and its differential regulation for the *STAR* and *ID3* mRNA expression in cultured granulosa cells, the present study supports the active role of ADRA2A involved in follicular development. To the best of our knowledge, our study represents the first to characterize the functionality and tissue expression of the ADRA2A in avian species.

### 4.1. Identification of ADRA2A in Chickens

Based on pharmacological studies, the α_2_-adrenoceptors have been divided into three subtypes α_2A_, α_2B_ and α_2C_, in mammals [52,53]. In chickens, only partial cDNA sequences (from the fourth to fifth transmembrane regions) of *ADRA2A* have been reported [54]. In the present study, for the first time, we cloned the complete cDNA of α_2A_-adrenoceptors gene (*ADRA2A)*. By alignment with the reported partial sequence of *ADRA2A*, the gene that we cloned shows the asparagine at position 214, whereas a tryptophan at this position is deduced [54]. Synteny analyses show that *ADRA2A* is orthologous to human *ADRA2A* and other species including mouse and turtle, implicating the conserved role of ADRA2A across species. 

In this study, we prove that ADRA2A can be activated by NE and EPI, indicating that it is functional in chickens. Similar to our findings, ADRA2A is reported to be activated by NE and EPI in mammals [17,19,55]. In the present study, functional studies reveal that ADAR2A is likely coupled to both G_s_ and G_q_ proteins, as evidenced by luciferase reporter assays and Western blot. The finding is in consistent with the observance that ADAR2A is reported to couple to G_s_ and G_q_ proteins [56,57,58,59,60]. However, the present study is different from the reports in mammals, where the ADRA2A is reported to couple to the G_i_ protein upon ligand activation and to inhibit the cAMP/PKA signaling pathway [19,61,62]. The differential G-protein coupling of ADRA2A among species reflects their diversified amino acids, and further, the differential role across species [62]. In the present study, cADAR2A is also found to be coupled to the MAPK/ERK signaling pathways and Ca^2+^ mobilization. Intracellular signaling may result from the involvement of βγ subunits in PLC activation [20,21,22], thus showing the typical α_2_-adrenoceptor signaling pathway supporting its wide physiological activities.

### 4.2. Tissue Distribution of ADRA2A in Chickens 

In this study, based on the RNA-Seq data sets deposited in the public database, we find that *cADRA2A* is widely distributed in CNS and peripheral tissues, including ovary, lung and adipose in the red jungle fowl. In chicken J-line, the mRNA expression of *cADRA2A* shows an abundance in thyroid and lung tissues. The difference in tissue distribution of *ADRA2A* mRNA between red jungle fowl and chicken J-line may result from varied developmental stage sampling. Within the CNS of red jungle fowl, *cADRA2A* shows a moderate expression level in brain regions including the hypothalamus, cerebellum and cerebrum. These findings are accordant to the previous studies in mammals and birds [17,62,63], in which α_2A_-adrenoceptors may play important roles in the CNS, including food intake [64], gonadotropin-releasing hormone (GnRH) and luteinizing hormone (LH) release [30,65,66], sedation [67] and consolidation of working memory [68]. In the present study, *cADRA2A* is highly expressed in peripheral tissues, including the thyroid, which is in line with the detection in mice that the α_2_-adrenoceptors have been reported to regulate thyroid hormone secretion [68,69,70].

### 4.3. Involvement of ADRA2A Signaling in Ovarian Follicular Growth and Differentiation

Follicular development is a complex process. The granulosa cell layer surrounding the growing oocyte undergoes dramatic morphological and physiological changes during the process of follicular development [71]. Before the follicle selection, the granulosa cells are mitotically active, which can proliferate and expand to keep pace with follicular growth, whereas granulosa cells are rapidly differentiated and become less proliferated after being selected into the preovulatory hierarchy [72,73]. In the present study, a high level of *ADRA2A* mRNA expression in 6–8 mm theca cells is detected. In coordination with the process, *ADRA2A* mRNA is also highly expressed in the GCs of 3–5 mm and 6–8 mm follicles and reaches its peak in the GCs of F5 follicles, thus supporting the hypothesis that ADRA2A signaling may be actively involved in follicular development. NE (10 μM, 16 h) can significantly increase the number of EdU-positive cells. The present study provides a molecular basis for the observance by EI-Habbak and his colleagues in 2008, such that continual injection of catecholamines brings about ovary growth [30]. Especially, the data reported in the present study that *ADRA2A* mRNA reaches its peak in the GCs of F5 follicles well support the observance that ovulation stops at a specific time, 14–16 h before ovulation for the antiadrenergic drugs’ injection effect [74]. Thus, in line with previous reports of NE and EPI being involved in regulation of ovarian follicular development, which is mediated by adrenoceptors, we show that this is further supported in chickens [9,74,75,76,77].

We found that NE also induces the mRNA expression of *STAR*. *STAR* encodes the key steroidogenic acute regulatory protein, which mediates the transport of cholesterol from the outer mitochondrial membrane to the inner [40,71]. In prehierarchical (6–8 mm) follicles, the granulosa cells are considered undifferentiated and steroidogenically inactive with weak expression of *STAR* [72]. After the follicle selection, *STAR* and other steroidogenic enzymes (e.g., *CYP11A1* and *CYP17A1*) are highly expressed in the differentiated granulosa cells and initiate the process of steroidogenesis [78]. In a way, the expression of *STAR* represents one of the changes in granulosa cells from an undifferentiated to a differentiated state. Hence, we suppose that NE may also be involved in the differentiation of granulosa cells during follicular development through ADRA2A signaling.

In our study, the treatment of NE (10 μM) for 24 h significantly decreases the expression of *ID3* in cultured prehierarchical granulosa cells (Figure 5G). ID3 is one of the ID (inhibitor of differentiation/DNA binding) family members, which include ID1, ID3, ID3 and ID4 proteins, belonging to the basic helix–loop–helix (bHLH) transcription factors family. The ID proteins, which lack the DNA binding domain, prevent the binding of bHLH transcription factors to the E-box site by forming heterodimers [79]. In the hen ovary, the selection of a single follicle per day into the preovulatory hierarchy occurs in 6–8 mm prehierarchical follicles, while the mechanism has yet to be defined. The most proximal marker for recently selected follicles is the increased expression of *FSHR* mRNA in the granulosa cells [71,78]. In undifferentiated granulosa cells, the TGF-β or activin A, could induce SMAD2 binding to SBEs, resulting in low levels of *FSHR* mRNA expression [80]. The effect of TGF-β or activin A could also be blocked by EGFRLs (epidermal growth factor ligands), which promote the expression of ID1, ID3 and ID4, thereby inhibiting the transcription of *FSHR* mRNA and maintaining the undifferentiated state in granulosa cells [81]. Based on the role of ID3 in regulating the transcription of *FSHR*, our finding that NE decreases the expression of *ID3* suggests that NE may be involved in the mRNA transcription of *FSHR* at the time of follicle selection. 

In summary, chicken *ADRA2A* has been cloned. Functional assays prove that it is functional for NE and EPI. In addition to the mRNA expression of *ADRA2A*, it has been found to be highly expressed in chicken tissues including thyroid, ovary, lung and adipose. The expression of *ADRA2A* mRNA has also been found to be highly expressed in granulosa cells from chicken developmental follicles including 6–8 mm follicles and F5 follicles. Together with the observance that NE stimulates the proliferation and differentiation of granulosa cells, the present study supports the active role of ADRA2A signaling along follicular development. Our data set a starting point to further elucidate the role of ADRA signaling in avian species.

## Figures and Tables

**Table 1:** Primers used in this study ^a^.

Gene/Construct Name	Sense/Antisense	Primer Sequence (5′–3′)	Size (bp)
Primers for constructing expression plasmids ^b^			
*cADRA2A*	Sense	CGGAATTCGCGCAGCGGGGTTGAT	1378
	Antisense	CGGAATTCCCCAGTGGGTCCTTC	
Primers for quantitative Real-time PCR assay			
*cSTAR*	Sense	CAGAGGGTTGGGAAGGACAC	204
	Antisense	CATACATGTGGGGCCGTTCT	
*cID3*	Sense	CAAGCTGAGCCAGGTGGAGATC	195
	Antisense	TGATGGAGGAGGCGTTAGTGACA	
*cβ-actin*	Sense	CCCAGACATCAGGGTGTGATG	123
	Antisense	GTTGGTGACAATACCGTGTTCAAT	

^a^ All primers were synthesized by BGI (China). ^b^ Restriction sites added in the 5’-end of the primers are underlined.

**Table 2:** EC_50_ values of NE and EPI in activating different signaling pathways of CHO cells expressing chicken ADRA2A.

Signaling Pathways	Peptides	EC_50_ (μM)
cAMP/PKA signaling pathway	NE	7.21 ± 1.15
	EPI	4.23 ± 1.36
MAPK/ERK signaling	NE	2.26 ± 0.97
	EPI	3.04 ± 0.64
Calcium mobilization	NE	6.74 ± 2.64
	EPI	3.73 ± 1.18

## Data Availability

Not applicable.
